# Circulating enterolactone and risk of breast cancer: a prospective study in New York

**DOI:** 10.1038/sj.bjc.6601893

**Published:** 2004-06-29

**Authors:** A Zeleniuch-Jacquotte, H Adlercreutz, R E Shore, K L Koenig, I Kato, A A Arslan, P Toniolo

**Affiliations:** 1 Department of Environmental Medicine, New York University School of Medicine, 650 First Avenue, Room 539, New York, NY 10016, USA; 2 New York University Cancer Institute, New York University School of Medicine, New York, NY 10016, USA; 3 Institute for Preventive Medicine, Nutrition and Cancer, Folkhälsan Research Center and Department of Clinical Chemistry, University of Helsinki, POB 63, FIN-00014, Finland; 4 Department of Pathology, Karmanos Cancer Institute, Wayne State University, 110 E Warren Ave., Detroit, MI 48201, USA; 5 Department of Obstetrics and Gynecology, New York University School of Medicine, New York, 550 First Avenue NB9E2, New York, NY 10016, USA

**Keywords:** breast cancer, enterolactone, lignan, phyto-oestrogen, prospective study

## Abstract

It has been proposed that phyto-oestrogens protect against breast cancer. Lignans are the main class of phyto-oestrogens in Western diets. We conducted a case–control study of breast cancer and serum levels of the main human lignan, enterolactone, nested within a prospective cohort study, the New York University Women's Health Study. Serum samples collected at enrollment and stored at −80°C were used. Among 14 275 participants, 417 incident breast cancer cases were diagnosed a median of 5.1 years after enrollment. Cohort members individually matched to the cases on age, menopausal status at enrollment, serum storage duration and, if premenopausal, day of menstrual cycle were selected as controls. No difference in serum enterolactone was observed between postmenopausal cases (median, 14.3 nmol l^−1^) and controls (14.5 nmol l^−1^), whereas premenopausal cases had higher levels (13.9 nmol l^−1^) than their matched controls (10.9 nmol l^−1^, *P*-value=0.01). In the latter group, the odds ratio for the highest *vs* the lowest quintile of enterolactone was 1.7 (95% confidence interval (CI), 0.8–3.4; *P*-value for trend=0.05) and after adjustment for known risk factors for breast cancer was 1.6 (95% CI, 0.7–3.4; *P*-value for trend=0.13). We observed a moderate positive correlation between serum enterolactone and serum sex hormone-binding globulin in postmenopausal women (*r*=0.29 in controls (*P*<0.001) and *r*=0.14 in cases (*P*=0.04)), but no correlation with oestrogens or androgens. These results do not support a protective role of circulating lignans, in the range of levels observed, in the development of breast cancer.

It has been suggested that phyto-oestrogens protect against hormone-dependent cancers, in particular breast cancer, through their weak agonist/antagonist oestrogen properties as well as their actions on oestrogen synthesis and bioavailability (Setchell *et al*, 1981; Adlercreutz *et al*, 1982; Adlercreutz, 1990; Rose, 1992). In addition, phyto-oestrogens have been reported to have antioxidant and antiproliferative properties which provide additional pathways through which they may reduce cancer risk (Adlercreutz and Mazur, 1997; Kurzer and Xu, 1997). The two main groups of phyto-oestrogens found in humans, the isoflavonoids and the lignans, are formed in the intestinal tract by transformation of plant precursors (isoflavone glycosides and plant lignans) by the microflora. Soy and soy products are the main sources of isoflavones, whereas lignans are found in oilseeds, in particular flaxseed, whole-grain products, nuts, berries, legumes, and some vegetables and fruits (Thompson *et al*, 1991; Adlercreutz and Mazur, 1997; Mazur, 1998; Horn-Ross *et al*, 2000).

A number of studies have focused on isoflavones and examined the association of intake of soy or soy protein with breast cancer risk, mostly in Asian populations (Peeters *et al*, 2003). Research on the association between the dietary intake of specific phyto-oestrogens, in particular lignans, and disease risk has been hampered, though, by the paucity of data on the amount of these compounds in individual foods, although the development of appropriate databases is underway (Horn-Ross *et al*, 2000; Kiely *et al*, 2003; Valsta *et al*, 2003). Dietary intake of foods known to be rich in lignan precursors has been shown to correlate positively with lignan plasma levels (Horner *et al*, 2002). Lignan intake, assessed by a 24-h dietary recall, also correlated positively with serum enterolactone (Kilkkinen *et al*, 2003), thus suggesting that levels of circulating enterolactone may provide a useful alternative approach to dietary questionnaires when assessing the effect of lignans on disease risk.

The association between circulating levels of the main lignan enterolactone and breast cancer risk was assessed in a case–control study nested within the New York University (NYU) Women's Health Study, a prospective cohort study of cancer, hormones, and environmental factors. The study focused exclusively on enterolactone based on the results of a preliminary study that examined the distribution and long-term reliability of serum measurements of the two main lignans, enterolactone and enterodiol, and of a number of isoflavonoid phyto-oestrogens. With the exception of enterolactone, phyto-oestrogen concentrations were below the detection limits for a large proportion of subjects, and the temporal reliability coefficients were low, indicating that our cohort was not well suited to study the effect of these compounds on disease risk (Zeleniuch-Jacquotte *et al*, 1998). As some of the mechanisms of action suggested for a protective effect of lignans are related to oestrogen synthesis and bioavailability, we also examined the correlation of serum enterolactone with circulating sex hormones and sex hormone-binding globulin (SHBG) in the group of postmenopausal women for whom these measurements were available (Zeleniuch-Jacquotte *et al*, 2004).

## MATERIALS AND METHODS

### The NYU Women's Health Study cohort

Between 1985 and 1991, the NYU Women's Health Study enrolled 14 275 healthy women aged 34–65 years at the Guttman Breast Diagnostic Institute, a breast cancer screening centre in New York City (Toniolo *et al*, 1991; Toniolo *et al*, 1995). Women who had been pregnant or taken hormonal medications in the 6 months preceding their visit were not eligible. At the time of enrollment, women were classified as postmenopausal if they reported no menstrual cycles in the previous 6 months, a total bilateral oophorectomy, or a hysterectomy without total oophorectomy prior to natural menopause and their age was 52 years or older. A total of 7054 participants (49.4%) were postmenopausal. After written informed consent was obtained, demographic, medical, anthropometric and reproductive data were collected through self-administered questionnaires. Dietary data were collected using a semiquantitative food frequency questionnaire based on the Block questionnaire used in NHANES II (Block *et al*, 1986, 1990). In total, 30 ml of non-fasting peripheral venous blood were drawn prior to breast examination. After blood drawing, tubes were kept covered at room temperature (21–25°C) for 15 min, then at 4°C for 60 min to allow clot retraction, and then centrifuged for 25 min. After centrifugation, serum samples were divided into 1 ml aliquots and immediately stored at −80°C for subsequent biochemical analyses. Up to 1991, women who returned for annual breast cancer screening were invited to contribute additional blood donations. Data on the use of oral contraceptives and hormone replacement therapy were collected in follow-up questionnaires mailed to participants in 1995 and in 1998.

### Nested case–control study of breast cancer

The current study was based on an ongoing case–control study of sex hormones and breast cancer. Cases were identified through active follow-up of the cohort by mailed questionnaires approximately every 2–4 years and telephone interviews for nonrespondents, as well as record linkage with state cancer registries in New York, New Jersey and Florida, and with the US National Death Index. A capture–recapture analysis estimated the ascertainment rate in our cohort to be 95% (Kato *et al*, 1999). Only incident cases (ie diagnosed at least 6 months after blood donation) of invasive breast cancer were included. Medical and pathology reports were requested to confirm the diagnosis.

Controls were selected at random among appropriate risk sets. The risk set for a case consisted of all women who were alive and free of cancer at the time of diagnosis of the case and who matched the case on menopausal status at the time of enrollment, age at entry (±6 months), date of enrollment (±3 months), and if premenopausal, day of menstrual cycle at the time of blood donation. For the present study of enterolactone and breast cancer, one control was selected at random between the two controls (or four for premenopausal women) selected for the study of sex hormones in order to limit study costs. In addition, the following exclusion criteria were applied to both cases and controls: (1) use of antibiotics in the 4 weeks preceding blood donation because such use has been shown to almost completely eliminate the formation of enterolactone from plant precursors in the gut (Borriello *et al*, 1985; Adlercreutz *et al*, 1986; Kilkkinen *et al*, 2002); (2) diagnosis with other cancer or cardiovascular disease in order to preserve the serum samples of these participants for the study of these diseases.

### Laboratory analyses

Enterolactone was measured by time-resolved fluoroimmunoassay (Adlercreutz *et al*, 1998) with slight modifications (Stumpf *et al*, 2001). For the recovery calculation, 15 *μ*l of 3H-oestradiol glucuronide (enterolactone glucuronide is not available) was added to the tubes containing 200 *μ*l of plasma and equilibrated for 30 min at room temperature. Then, 200 *μ*l of acetate buffer 0.1 M pH 5.0 containing 2 U ml^−1^ sulphatase and 0.2 U ml^−1^ *β*-glucuronidase were added. After mixing, the tubes were incubated overnight at 37°C. After hydrolysis, the free enterolactone was extracted twice with 1.5 ml of ethyl ether. The separation of the phases was carried out by freezing the water phase. After evaporation of the ether, 200 *μ*l of the TR-FIA assay buffer was added and a 20 *μ*l aliquot taken for calculation of recovery and two 20 *μ*l aliquots used for the time resolved fluoroimmunoassay. Samples from a case and her matched control were always assayed in the same batch. The laboratory analyses were performed blind, and all of the batches were analysed with two quality control samples used throughout the whole procedure, and three samples used for the immunoassay step only. Two samples from a common pool that were labelled to preclude their identification were interspersed every 11 matched sets to assess the laboratory precision. The mean of the within-batch coefficient of variation for the seven batches was 8.9%.

### Statistical methods

Enterolactone measurements were log-transformed to reduce departure from the normal distribution. To test for differences in enterolactone levels (and other continuous variables) between case and control subjects, we used a mixed-effects regression model to take into account the matched design (Liang and Zeger, 1986). *P*-values to compare proportions between case and control subjects were generated using the conditional logistic regression model.

To compute odds ratios (ORs), serum measurements were categorised into quintiles separately for pre- and postmenopausal subjects, using the frequency distribution of the cases and the controls combined. The data were analysed using conditional logistic regression (Breslow and Day, 1980). Odds ratios were computed relative to the lowest quintile. Likelihood ratio tests were used to assess the significance of overall associations, linear trends and deviations from linearity. Reported trend test *P*-values correspond to enterolactone variables treated as ordered categorical variables. Analyses were also performed on the continuous variables. Analyses were conducted using the SAS Release 8.02 and EGRET statistical packages. All *P*-values are two-sided.

## RESULTS

A total of 461 incident breast cancer cases diagnosed prior to 1 January 1995 had been identified by the start date of the study, 1 September 1998. A total of 38 cases were excluded from the present study for the following reasons: diagnosis of other cancer prior or subsequent to diagnosis (*n*=24), diagnosis of coronary heart disease (*n*=3), and use of antibiotics in the 4 weeks prior to study enrollment (*n*=11). Six additional cases were excluded because the volume of serum was insufficient for the assay. As a result, 417 cases are included in this report. Nine participants became cases after being selected as controls and are included both as cases and controls in the appropriate matched sets (Robins *et al*., 1986). Pathology reports were obtained for 349 cases (84%) and the diagnoses of 59 additional cases (14%) were confirmed through Tumour Registries.

Table 1

**Table 1:** Study subject characteristics by menopausal status at blood donation

	**Premenopausal women**		**Postmenopausal women**	
	**Cases (*n*=189)**	**Controls (*n*=189)**	**Cases (*n*=228)**	**Controls (*n*=228)**
Age at blood donation, median (5–95th percentiles)	44.2 (36.6–51.7)	44.2 (36.3–52.0)	58.8 (52.5–64.8)	58.8 (52.5–65.0)
Age at diagnosis, median (5–95th percentiles)	49.1 (40.1–57.0)		64.2 (55.8–70.6)	
Age at menarche, median (5–95th percentiles)	12 (10–15)	12 (10–16)	13 (10–15)	12 (10–15)
Nulliparous, *n* (%)	73 (39%)	58 (31%)	57 (25%)	47 (21%)
Age at first full-term pregnancy, median (5–95th percentiles)^b^	25 (19–39)	24 (18–36)	25 (19–35)	24 (19–34)
History of breast cancer in first-degree relatives, *n* (%)^c^	51 (27%)	25 (13%)	43 (19%)	51 (22%)
Total oophorectomy, *n* (%)	0	0	29 (13%)	25 (11%)
Ever used oral contraceptives, *n* (%)	91/164 (55%)	105/168 (63%)	44/198 (22%)	32/205 (16%)
Ever used hormone replacement therapy, *n* (%)^d^	14/164 (9%)	26/168 (15%)	32/198 (16%)	30/205 (15%)
Height (cm), median (5–95th percentiles)	163 (152–175)	163 (152–173)	163 (152–173)	163 (152–170)
Weight (kg), median (5–95th percentiles)^e^	61.3 (49.0–84.0)	61.3 (49.0–89.0)	68.1 (51.8–89.9)	63.6 (49.0–88.5)
Body mass index (kg m^−2^), median (5–95th percentiles)^f^	23.0 (18.5–31.3)	23.6 (19.0–32.5)	25.7 (20.2–33.0)	24.3 (19.7–32.5)
Energy intake (kcal), median (5–95th percentiles)	1319 (720–2475)	1359 (792–2510)	1427 (807–2723)	1491 (760–2549)
Percent energy from fat intake, median (5–95th percentiles)	43.0 (30.2–51.7)	42.2 (30.7–52.3)	38.8 (28.2–50.7)	39.2 (28.2–48.2)
Daily dietary fibre intake (g), median (5–95th percentiles)	11.2 (4.5–23.0)	10.9 (1.3–42.7)	14.5 (1.9–51.6)	14.3 (1.3–52.8)

a Unless specified, *P*-values were greater than 0.10.

b *P*=0.08 in premenopausal women.

c *P*=0.001 in premenopausal women.

d *P*=0.05 in premenopausal women.

e *P*=0.01 in postmenopausal women.

f *P*=0.09 in postmenopausal women.

presents the characteristics of the study subjects by menopausal status. The median age at blood donation was 44 years in premenopausal women, and 59 years in postmenopausal women, and the overall median lagtime between blood donation and diagnosis was 5.1 years (range, 6 months to 9.5 years). Overall, the proportion of nulliparous women was higher among cases (31%) than among controls (25%, *P*=0.05). Although no longer statistically significant, this difference was observed in both pre- and postmenopausal women. Among women premenopausal at blood donation, cases tended to be older at first full-term pregnancy (*P*=0.08), reported more often a family history of breast cancer (*P*=0.001) and were less likely to have used hormone replacement therapy than controls (*P*=0.05). Among postmenopausal women, cases tended to have a higher weight (*P*=0.01) and body mass index (*P*=0.09) than controls.

Table 2

**Table 2:** Descriptive statistics of serum enterolactone levels in cases and controls

**Enterolactone (nmol l^−1^)**	**Cases**	**Controls**	***P*-value^a^**
*All women (417 matched sets)*			
Arithmetic mean (s.d.)	18.5 (16.1)	17.2 (15.7)	0.04
Median (5–95th percentiles)	14.3 (2.0–49.6)	12.8 (1.3–51.1)	
			
*Premenopausal women (189 matched sets)*			
Mean (s.d.)	18.3 (17.0)	15.1 (14.2)	0.01
Median (5–95th percentiles)	13.9 (2.5–47.0)	10.9 (1.3–42.7)	
			
*Postmenopausal women (228 matched sets)*			
Mean (s.d.)	18.6 (15.4)	18.9 (16.7)	0.51
Median (5–95th percentiles)	14.5 (1.9–51.6)	14.3 (1.3–52.8)	

a *P*-values are from the mixed-effects regression models, controlling for matching factors.

reports serum levels of enterolactone. Overall, levels were higher in cases (median, 14.3 nmol l^−1^) than in controls (12.8 nmol l^−1^, *P*=0.04). When levels were examined according to menopausal status, no difference was observed between postmenopausal cases and controls, whereas premenopausal cases had higher levels (median, 13.9 nmol l^−1^) than their matched controls (10.9 nmol l^−1^, *P*=0.01).

Table 3

**Table 3:** Odds ratios (95% confidence intervals) and logistic regression coefficients (standard error) for breast cancer associated with premenopausal serum levels of enterolactone

	**Cases, *n* (%)**	**Controls, *n* (%)**	**Simple odds ratios (95% CI)^a^**	**Multivariate odds ratios (95% CI)^b^**
*Serum enterolactone (nmol l*^−*1*^) *quintiles*				
⩽4.98	32 (17%)	45 (24%)	1.0	1.0
4.99–9.95	34 (18%)	41 (22%)	1.3 (0.7–2.5)	1.3 (0.7–2.7)
9.96–16.04	37 (20%)	38 (20%)	1.4 (0.7–2.8)	1.3 (0.6–2.7)
16.05–24.09	44 (23%)	32 (17%)	1.9 (1.0–3.6)	1.7 (0.9–3.3)
⩾24.10	42 (22%)	33 (17%)	1.7 (0.8–3.4)	1.6 (0.7–3.4)
*P*-value for trend			0.05	0.13
ln (enterolactone),				
*β* (s.e.)			0.252 (0.112)	0.218 (0.121)
*P*-value			0.02	0.07

a Controlled for matching factors.

b Controlled for matching factors and adjusted for age at menarche, family history of breast cancer in mother or sisters, nulliparity and age at first full-term pregnancy (< 23, 23–25, 26–29, ⩾30, nulliparous), ln(height), ln(body mass index).

reports ORs for breast cancer associated with quintiles of enterolactone in premenopausal women. In the conditional model controlling only for matching factors, a trend of increasing risk with increasing level of enterolactone was observed (*P*=0.05). The OR was 1.7 (95% CI, 0.8–3.4) in the highest quintile, which was slightly reduced to 1.6 (0.7–3.4) after adjusting for the following known risk factors: age at menarche, family history of breast cancer, parity, age at first full time pregnancy, height and body mass index (*P* for trend=0.13). Adjusting further for use of oral contraceptives and hormone replacement therapy did not materially affect the OR but reduced the number of matched sets available for analysis to 139 (74% of total) and are therefore not presented. Similar results were obtained when log-transformed enterolactone was used on the continuous scale. Table 4

**Table 4:** Odds ratios (95% confidence intervals) and logistic regression coefficients (standard error) for breast cancer associated with postmenopausal serum levels of enterolactone

	**Cases, *n* (%)**	**Controls, *n* (%)**	**Simple odds ratios (95% CI)^a^**	**Multivariate odds ratios (95% CI)^b^**
*Serum enterolactone (nmol l*^−*1*^)				
⩽5.40	43 (19%)	48 (21%)	1.0	1.0
5.41–11.41	45 (20%)	46 (20%)	1.1 (0.6–2.0)	1.3 (0.7–2.5)
11.42–19.30	48 (21%)	44 (19%)	1.2 (0.7–2.2)	1.2 (0.6–2.3)
19.31–29.07	45 (20%)	46 (20%)	1.1 (0.6–1.9)	1.3 (0.7–2.3)
⩾29.08	47 (21%)	44 (19%)	1.2 (0.7–2.3)	1.0 (0.5–2.1)
*P*-value for trend			0.58	0.95
				
ln (enterolactone), *β* (s.e.)			0.061 (0.092)	0.044 (0.101)
*P*-value			0.50	0.71

a Controlled for matching factors.

b Controlled for matching factors and adjusted for age at menarche, family history of breast cancer in mother or sisters, nulliparity and age at first full-term pregnancy (< 23, 23–25, 26–29, ⩾30, nulliparous), ln(height), ln(body mass index).

shows the results among postmenopausal women. No association of serum enterolactone with breast cancer risk was observed in this group.

There was no evidence of correlation between enterolactone and oestrogens or androgens: all correlation coefficients were less than 0.10 (Table 5

**Table 5:** Spearman correlation coefficients of serum enterolactone with circulating sex hormones and SHBG in postmenopausal women

	**Cases (*n*=188)**		**Controls (*n*=181)**	
	**Unadjusted**	**Adjusted^a^**	**Unadjusted**	**Adjusted^a^**
Oestradiol	0.04	0.07	−0.09	−0.07
Oestrone	0.06	0.09	−0.06	−0.05
Testosterone	0.12*	0.15**	0.02	0.03
Androstenedione	−0.03	−0.02	−0.10	−0.07
DHEAS	0.02	0.04	−0.14*	−0.12
SHBG	0.14**	0.13*	0.29***	0.26***

a Adjusted for body mass index.

* *P*< 0.10,

** *P*< 0.05,

*** *P*< 0.001. All other *P*-values >0.10.

). However, we observed a positive correlation between enterolactone and SHBG, which was stronger in controls (0.29, *P*<0.001) than in cases (0.14, *P*=0.04). This positive correlation persisted after adjusting for body mass index, a strong predictor of circulating SHBG: the partial correlation coefficients were 0.26 (*P*<0.001) in controls and 0.13 in cases (*P*=0.09).

## DISCUSSION

Contrary to our hypothesis, we did not observe an inverse association between serum levels of enterolactone and risk of breast cancer. With 417 cases, the study had 80% power to detect an odds ratio of 0.59 in the highest *vs* the lowest quartile, that is, a 41% reduction in risk. Although we cannot rule out an association of smaller magnitude, we believe that the lack of inverse association between enterolactone and breast cancer risk in our study is not due to lack of statistical power because of the absence of any trend among postmenopausal women and the marginally significant positive trend observed among premenopausal women.

We also considered whether error in exposure measurement could explain these results, since enterolactone was measured at a single point in time whereas the true exposure of interest is long-term average concentration of enterolactone. However, in a preliminary study we have shown that, in our cohort subjects, circulating enterolactone is fairly stable over a 2-year period of time, as indicated by a reliability coefficient of 0.55 (Zeleniuch-Jacquotte *et al*, 1998). In addition, because the study was based on serum samples obtained prior to diagnosis of disease, the error in measurement would be expected to be nondifferential with respect to case–control status and therefore very unlikely to result in a change of direction of the exposure–disease association in premenopausal women (Kelsey *et al*, 1996).

Antibiotic use, through its action on the microflora of the gut, greatly reduces circulating levels of enterolactone (Kilkkinen *et al*, 2002). Women who reported taking antibiotics in the 4 weeks preceding blood donation were excluded, but recent data show that serum enterolactone concentrations could be significantly lower among subjects who use oral antimicrobials up to 12–16 months before serum sampling, as compared to nonusers (Kilkkinen *et al*, 2002). Some women, both among cases and controls, with usually high serum enterolactone levels may have been misclassified as having low levels if they had taken antibiotics in the 1–16 months prior to enrollment. However, it is again very unlikely that such misclassification would result in a spurious positive association among premenopausal women since we used prediagnostic sera.

Few studies have been published to date on the association between biological levels of lignans and breast cancer risk. Three studies reported on the urinary excretion of phyto-oestrogens including lignans. In an individually matched case–control study in Western Australia (144 cases), Ingram *et al* (1997) reported a significant trend of reduced risk with increasing urinary excretion levels of both equol and enterolactone. Results were not presented separately for pre- and postmenopausal women, but the authors noted that similar trends were observed within each group. A population-based case–control study among Chinese women in Shanghai (250 cases) reported a reduced risk with increasing urinary excretion of total isoflavonoids and total lignans, which was present in both pre- and postmenopausal women, although stronger in premenopausal women (Dai *et al*, 2002). The only prospective study that examined urinary phyto-oestrogen excretion was a case–control study (88 cases and 268 controls) nested within a cohort of postmenopausal Dutch women participating in a breast cancer screening study (den Tonkelaar *et al*, 2001). Elevated urinary genistein was weakly and nonsignificantly associated with a risk reduction, whereas elevated urinary enterolactone was weakly and nonsignificantly associated with increased risk.

Three studies have reported on the association of circulating levels of lignans with breast cancer risk. A population-based case–control study examined serum levels of enterolactone in 194 cases and 208 controls in Eastern Finland (Pietinen *et al*, 2001). A significant inverse association with risk was observed. Results were similar in pre- and postmenopausal women. In a case–control study nested in three population-based cohorts in northern Sweden, there was no significant association of breast cancer risk with serum enterolactone but further analyses showed a U-shaped relationship with an increase in risk at both very low (<5.5 nmol l^−1^), and very high (>39.1 nmol l^−1^) concentrations (Hultén *et al*, 2002). Analyses stratifying by cohort showed that the increase in risk with high levels of enterolactone was observed in the two cohorts with only incident cases and a high proportion of premenopausal women (54%), whereas in the third cohort consisting mostly of older women (85% postmenopausal) from a mammary screening project with mostly prevalent cases, a slight, nonsignificant reduction in risk was observed with high plasma enterolactone levels. Another nested case–control study, conducted in Finland, showed no association, overall or within menopausal groups (Kilkkinen *et al*, 2004).

It is noteworthy that all three retrospective case–control studies (Ingram *et al*, 1997; Pietinen *et al*, 2001; Dai *et al*, 2002) reported an inverse association of enterolactone with risk, whereas three of the prospective studies reported no or weak positive associations and one reported a U-shaped association with risk. It is unlikely that the results from the case–control studies could be explained by qualitative dietary modifications of the cases because one would expect that women changing their diet in response to suspicion or diagnosis of breast cancer would switch to ‘healthy diets’ usually associated with increased enterolactone intake. However, whereas controls contributed biological samples at their convenience, collection of samples from cases occurred before admission to hospital for surgery (Ingram *et al*, 1997), the morning of, or 1–2 days before surgery (Dai *et al*, 2002), or at a referral clinical examination for breast symptom or suspected breast lump (Pietinen *et al*, 2001). It is thus possible that cases, in these stressful circumstances, reduced their overall intake of food, including their intake of lignan-rich foods, resulting in lower circulating or urinary enterolactone levels. In addition, in two of the studies, it is possible that control subjects who agreed to participate were self-selected for health-conscious behaviors, including eating a healthy diet rich in lignan precursors: the control participation rate was 72% in the Finnish study (Pietinen *et al*, 2001) and 33% in the Australian study (Ingram *et al*, 1997).

Studies quantifying the intakes of specific phyto-oestrogens using a food frequency questionnaire and a nutrient database may shed additional light on the role of these compounds. Only one such study has been published to date in relation to breast cancer. The study was a case–control study in the San Francisco Bay Area that included 1326 cases and 1657 controls from various non-Asian ethnic backgrounds (Horn-Ross *et al*, 2001). Intake of total phyto-oestrogens was not associated with altered risk, including in analyses stratified by menopausal status or ethnic group. Analyses by intake of seven specific phyto-oestrogenic compounds, including lignans (matairesinol, secoisolariciresinol, and total lignans) were also negative.

Enterolactone at plasma levels that could easily be achieved in humans (400 nmol l^−1^) has been shown to inhibit both formation and growth of breast tumours in rats (Saarinen *et al*, 2002). The mechanisms that have been suggested for a protective role of lignans against breast cancer include antioxidant effects (Kitts *et al*, 1999), competitive binding with oestrogen receptors (Martin *et al*, 1978; Adlercreutz *et al*, 1992) and oestrogen synthesis and bioavailability (Adlercreutz, 1998). In cell culture systems, enterolactone has been shown to be a moderate inhibitor of aromatase, which is involved in the peripheral conversion of androgens to oestrogens in postmenopausal women (Adlercreutz *et al*, 1993; Wang *et al*, 1994). However, we did not observe an association between circulating enterolactone and oestrogens in our study. Lignans have also been shown to stimulate SHBG synthesis in the liver (Adlercreutz *et al*, 1987, 1992). Adlercreutz *et al* (1992) reported a significant positive correlation between the urinary excretion of enterolactone and total lignans with plasma SHBG in 30 postmenopausal women. In agreement with this study, we observed a moderate positive correlation between serum enterolactone and serum SHBG. Although it is therefore possible that lignans increase SHBG levels, this effect did not translate into a reduction in risk of breast cancer in our study.

Phyto-oestrogens could also affect the risk of breast cancer in premenopausal women through changes in the menstrual cycle length. Longer cycles have been shown to be associated with a reduced risk (Olsson *et al*, 1983; LaVecchia *et al*, 1985), as would be expected under the hypothesis that cumulative frequency of ovulatory cycles is a primary determinant of risk (Henderson *et al*, 1985). Both daily supplementation with 10 g of flaxseed, which is very rich in lignans (Phipps *et al*, 1993), and a diet rich in soy protein (Cassidy *et al*, 1994), a source of isoflavones, have been reported to increase the length of the menstrual cycle, although not in all studies (Wu *et al*, 2000; Maskarinec *et al*, 2002). However, whereas this increase appeared to be due to an increase in follicular phase length in the case of the soy protein diet (Cassidy *et al*, 1994), flaxseed supplementation was associated with an increase in luteal phase length (Phipps *et al*., 1993). As breast cell division is low during the follicular phase but high during the luteal phase with a peak at days 23–25 (Masters *et al*, 1977; Meyer, 1977; Ferguson and Anderson, 1981; Going *et al*, 1988; Potten *et al*, 1988), an increase in length of the luteal phase could contribute to an increase in risk of breast cancer. Such a mechanism would be consistent with our results as well as those of the den Tonkelaar study (2001). However, these diet supplementation studies were small in size and their results should be regarded as preliminary. In addition, it is not clear that the generally moderate biological levels of enterolactone in both our study and the den Tonkelaar study would have a noticeable impact on menstrual cycle length. Circulating levels of enterolactone following flaxseed supplementation are likely to be much higher than in our study (Morton *et al*, 1994; Tarpila *et al*, 2002).

In three of the prospective studies, an increase of breast cancer risk was observed at high levels of enterolactone, although the results were not significant in the Dutch study and only marginally significant in our study. These results suggest a need for further studies, both experimental and epidemiological, to better understand the overall impact of lignans in general, and enterolactone in particular, on the development of breast cancer. It also suggests caution against large dietary intake of flaxseed or use of flaxseed supplements for prevention: flaxseed is the oilseed with the richest amount of lignan precursors, and flaxseed supplementation can more than double serum enterolactone concentration (Tarpila *et al*, 2002).

## References

[bib1] Adlercreutz H, Mazur W (1997) Phyto-oestrogens and western diseases. Ann Med 29: 95–120 9187225 10.3109/07853899709113696

[bib2] Adlercreutz H (1990) Western diet and western diseases: some hormonal and biochemical mechanisms and associations. Scand J Clin Lab Invest 50(Supplement 201): 3–23 2173856

[bib3] Adlercreutz H (1998) Evolution, nutrition, intestinal microflora, and prevention of cancer: a hypothesis. Proc Soc Exp Biol Med 217: 241–246 9492331 10.3181/00379727-217-44228

[bib4] Adlercreutz H, Bannwart C, Wähälä K, Mäkelä T, Brunow G, Hase T, Arosemena PJ, Kellis JT, Vickery LE (1993) Inhibition of human aromatase by mammalian lignans and isoflavonoid phytoestrogens. J Steroid Biochem Mol Biol 44: 147–153 8382517 10.1016/0960-0760(93)90022-o

[bib5] Adlercreutz H, Fotsis T, Bannwart C, Wähälä K, Mäkelä T, Brunow G, Hase T (1986) Determination of urinary lignans and phytoestrogen metabolites, potential antiestrogens and anticarcinogens, in urine of women on various habitual diets. J Steroid Biochem 25: 791–797 3027456 10.1016/0022-4731(86)90310-9

[bib6] Adlercreutz H, Fotsis T, Heikkinen R, Dwyer J, Woods M, Goldin BR, Gorbach SL (1982) Excretion of the lignans enterolactone and enterodiol and of equol in omnivorous and vegetarian postmenopausal women and in women with breast cancer. Lancet 2: 1295–1299 6128595 10.1016/s0140-6736(82)91507-0

[bib7] Adlercreutz H, Höckerstedt K, Bannwart C, Bloigu S, Hämäläinen E, Fotsis T, Ollus A (1987) Effect of dietary components including lignans and phytoestrogens, on enterohepatic circulation and liver metabolism of estrogens and on sex hormone binding globulin (SHBG). J Steroid Biochem 27: 1135–1144 2826899 10.1016/0022-4731(87)90200-7

[bib8] Adlercreutz H, Mousavi Y, Clark J, Höckerstedt K, Hämäläinen E, Wähälä K, Mäkelä T, Hase T (1992) Dietary phytoestrogens and cancer: *in vitro* and *in vivo* studies. J Steroid Biochem Mol Biol 41: 331–337 1314077 10.1016/0960-0760(92)90359-q

[bib9] Adlercreutz H, Wang G, Lapcík O, Hampl R, Wähälä K, Mäkelä K, Lusa K, Talme M, Mikola H (1998) Time-resolved fluorimmunoassay for plasma enterolactone. Anal Biochem 265: 208–215 9882394 10.1006/abio.1998.2886

[bib10] Block G, Hartman A, Dresser C, Carroll M, Gannon J, Gardner L (1986) A data-based approach to diet questionnaire design and testing. Am J Epidemiol 124: 453–469 3740045 10.1093/oxfordjournals.aje.a114416

[bib11] Block G, Woods M, Potosky A, Clifford C (1990) Validation of a self-administered diet history questionnaire using multiple diet records. J Clin Epidemiol 43: 1327–1335 2254769 10.1016/0895-4356(90)90099-b

[bib12] Borriello SP, Setchell KD, Axelson M, Lawson AM (1985) Production and metabolism of lignans by the human faecal flora. J Appl Bacteriol 58: 37–43 2984153 10.1111/j.1365-2672.1985.tb01427.x

[bib13] Breslow NE, Day NE (1980) Statistical Methods in Cancer Research, Vol 1. International Agency for Research on Cancer: Lyon

[bib14] Cassidy A, Bingham S, Setchell KD (1994) Biological effects of a diet of soy protein rich in isoflavones on the menstrual cycle of premenopausal women. Am J Clin Nutr 60: 333–340 8074062 10.1093/ajcn/60.3.333

[bib15] Dai Q, Franke AA, Jin F, Shu XO, Hebert JR, Custer LJ, Cheng J, Gao YT, Zheng W (2002) Urinary excretion of phytoestrogens and risk of breast cancer among Chinese women in Shanghai. Cancer Epidemiol Biomarkers Prev 11: 815–821 12223424

[bib16] den Tonkelaar I, Keinan-Boker L, Van't Veer P, Arts CJM, Adlercreutz H, Thijssen JHH, Peeters PHM (2001) Urinary phytoestrogens and postmenopausal breast cancer risk. Cancer Epidemiol Biomarkers Prev 10: 223–228 11303591

[bib17] Ferguson DJP, Anderson TJ (1981) Morphologic evaluation of cell turnover in relation to the menstrual cycle in the ‘resting’ human breast. Br J Cancer 44: 177–181 7272186 10.1038/bjc.1981.168 PMC2010743

[bib18] Going JJ, Anderson TJ, Battersby S, MacIntyre CC (1988) Proliferative and secretory activity in human breast during natural and artificial menstrual cycles. Am J Pathol 130: 193–204 3337211 PMC1880536

[bib19] Henderson BE, Ross RK, Judd H, Krailo M, Pike MC (1985) Do regular ovulatory cycles increase breast cancer risk? Cancer 1206–1208 10.1002/1097-0142(19850901)56:5<1206::aid-cncr2820560541>3.0.co;2-9 4016708

[bib20] Horner NK, Kristal AR, Prunti J, Skor HE, Potter JD, Lampe JW (2002) Dietary determinants of plasma enterolactone. Cancer Epidemiol Biomarkers Prev 11: 121–126 11815409

[bib21] Horn-Ross PL, John EM, Lee M, Stewart SL, Koo J, Sakoda LC, Shiau AC, Goldstein J, Davis P, Perez-Stable EJ (2001) Phytoestrogen consumption and breast cancer risk in a multiethnic population. Am J Epidemiol 154: 434–441 11532785 10.1093/aje/154.5.434

[bib22] Horn-Ross P, Barnes S, Lee M, Coward L, Mandel JE, Koo J, John EM, Smith M (2000) Assessing phytoestrogen exposure in epidemiologic studies: development of a database (United States). Cancer Causes Control 11: 289–298 10843440 10.1023/a:1008995606699

[bib23] Hultén K, Winkvist A, Lenner P, Johansson R, Adlercreutz H, Hallmans G (2002) An incident case-referent study on plasma enterolactone and breast cancer risk. Eur J Nutr 41: 168–176 12242585 10.1007/s00394-002-0373-3

[bib25] Ingram D, Sanders K, Kolybaba M, Lopez D (1997) Case–control study of phyto-oestrogens and breast cancer. Lancet 350: 990–994 9329514 10.1016/S0140-6736(97)01339-1

[bib26] Kato I, Toniolo P, Koenig K, Kahn A, Schymura M, Zeleniuch-Jacquotte A (1999) Comparison of active and cancer registry-based follow-up for breast cancer in a prospective cohort study. Am J Epidemiol 149: 372–378 10025481 10.1093/oxfordjournals.aje.a009823

[bib27] Kelsey JL, Whittemore AS, Evans AS, Thompson WD (1996) Methods in Observational Epidemiology. Oxford University Press: New York, Oxford

[bib28] Kiely M, Faughnan M, Wahala K, Brants H, Mulligan A (2003) Phyto-oestrogen levels in foods: the design and construction of the VENUS database. Br J Nutr 89(Suppl 1): S19–S23 12725652 10.1079/BJN2002792

[bib29] Kilkkinen A, Pietinen P, Klaukka T, Virtamo J, Korhonen P, Adlercreutz H (2002) Use of oral antimicrobials decreases serum enterolactone concentration. Am J Epidemiol 155: 472–477 11867359 10.1093/aje/155.5.472

[bib30] Kilkkinen A, Valsta LM, Virtamo J, Stumpf K, Adlercreutz H, Pietinen P (2003) Intake of lignans is associated with serum enterolactone concentration in Finnish men and women. J Nutr 133: 1830–1833 12771325 10.1093/jn/133.6.1830

[bib31] Kilkkinen A, Virtamo J, Vartiainen E, Sankila R, Virtanen MJ, Adlercreutz H, Pietinen P (2004) Serum enterolactone concentration is not associated with breast cancer risk in a nested case–control study. Int J Cancer 108: 277–280 14639615 10.1002/ijc.11519

[bib32] Kitts DD, Yuan J-M, Wijewickreme AN, Thompsom LU (1999) Antioxidant activity of the flaxseed lignan secoisolariciresinol diglycoside and its mammalian lignan metabolites enterodiol and enterolactone. Mol Cell Biochem 202: 91–100 10705999 10.1023/a:1007022329660

[bib33] Kurzer MS, Xu X (1997) Dietary phytoestrogens. Annu Rev Nutr 17: 353–381 9240932 10.1146/annurev.nutr.17.1.353

[bib34] LaVecchia C, Decarli A, di Pietro S, Franceschi S, Negri E, Parazzini F (1985) Menstrual cycle patterns and the risk of breast disease. Eur J Cancer Clin Oncol 21: 417–422 4007015 10.1016/0277-5379(85)90030-6

[bib35] Liang IK, Zeger SL (1986) Longitudinal data analysis using generalized linear models. Biometrika 73: 13–22

[bib36] Martin PM, Horwitz KB, Ryan DS, McGuire WL (1978) Phytoestrogen interaction with estrogen receptors in human breast cancer cells. Endocrinology 103: 1860–1867 570914 10.1210/endo-103-5-1860

[bib37] Maskarinec G, Williams AE, Inouye JS, Stanczyk FZ, Franke A (2002) A randomized isoflavone intervention among premenopausal women. Cancer Epidemiol Biomarkers Prev 11: 195–201 11867507

[bib38] Masters JRW, Drife JO, Scarisbrick JJ (1977) Cyclic variations of DNA synthesis in human breast epithelium. J Natl Cancer Inst 58: 1263–1265 853524 10.1093/jnci/58.5.1263

[bib39] Mazur W (1998) Phytoestrogen content in foods. Baillieres Clin Endocrinol Metab 12: 729–742 10384822 10.1016/s0950-351x(98)80013-x

[bib40] Meyer JS (1977) Cell proliferation in normal human breast ducts, fibroadenomas, and other duct hyperplasias, measured by nuclear labeling with tritiated thymidine. Hum Pathol 8: 67–81 844855 10.1016/s0046-8177(77)80066-x

[bib41] Morton MS, Wilcox G, Wahlqvist ML, Griffiths K (1994) Determination of lignans and isoflavonoids in human female plasma following dietary supplementation. J Endocrinol 142: 251–259 7930998 10.1677/joe.0.1420251

[bib42] Olsson H, Landin-Olsson M, Gullberg B (1983) Retrospective assessment of menstrual cycle length in patients with breast cancer, in patients with benign breast disease, and in women without breast disease. J Natl Cancer Inst 70: 17–20 6571912

[bib43] Peeters PHM, Keinan-Boker L, van der Schouw YT, Grobbee DE (2003) Phytoestrogens and breast cancer risk. Review of the epidemiological evidence. Breast Cancer Res Treat 77: 171–183 12602916 10.1023/a:1021381101632

[bib44] Phipps WR, Martini MC, Lampe JW, Slavin JL, Kurzer M (1993) Effect of flax seed ingestion on the menstrual cycle. J Clin Endocrinol Metab 77: 1215–1219 8077314 10.1210/jcem.77.5.8077314

[bib45] Pietinen P, Stumpf K, Männistö S, Kataja V, Uusitupa M, Adlercreutz M (2001) Serum enterolactone and risk of breast cancer: a case–control study in Eastern Finland. Cancer Epidemiol Biomarkers Prev 10: 339–344 11319174

[bib46] Potten CS, Watson RJ, Williams GT, Tickle S, Roberts SA, Harris M, Howell A (1988) The effect of age and menstrual cycle upon proliferative activity of the normal human breast. Br J Cancer 58: 163–170 3166907 10.1038/bjc.1988.185 PMC2246757

[bib47] Robins J, Gail MH, Lubin JH (1986) More on ‘Biased selection of controls for case–control analyses of cohort studies’. Biometrics 42: 293–299 3741971

[bib48] Rose DP (1992) Dietary fiber, phytoestrogens, and breast cancer. Nutrition 8: 47–51 1314118

[bib49] Saarinen N, Huovinen R, Warri A, Makela S, Valentin-Blasini L, Sjoholm R, Ammala J, Lehtila R, Eckerman C, Collan Y, Santti RS (2002) Enterolactone inhibits the growth of 7,12-dimethylbenz(a)anthracene-induced mammary carcinomas in the rat. Mol Cancer Ther 1: 869–876 12492120

[bib50] Setchell KD, Lawson AM, Borriello SP, Harkness R, Gordon H, Morgan DML, Kirk DN, Adlercreutz H, Anderson LC, Axelson M (1981) Lignan formation in man-microbial involvement and possible roles in relation to cancer. Lancet 2: 4–7 6113409 10.1016/s0140-6736(81)90250-6

[bib51] Stumpf K, Uehara M, Nurmi T, Adlercreutz H (2001) Changes in the time-resolved fluoroimmunoassay of plasma enterolactone. Anal Biochem 284: 153–157 10.1006/abio.2000.4655 10933868

[bib52] Tarpila S, Aro A, Salminen I, Tarpila A, Kleemola P, Akkika J, Adlercreutz H (2002) The effect of flaxseed supplementation in processed foods on serum fatty acids and enterolactone. Eur J Clin Nutr 56: 157–165 11857049 10.1038/sj.ejcn.1601298

[bib53] Thompson LU, Robb P, Serraino M, Cheung F (1991) Mammalian lignan production from various foods. Nutr Cancer 16: 43–52 1656395 10.1080/01635589109514139

[bib54] Toniolo PG, Pasternack BS, Shore RE, Sonnenschein EG, Koenig KL, Rosenberg C, Strax P, Strax S (1991) Endogenous hormones and breast cancer: a prospective cohort study. Breast Cancer Res Treat 18: S23–S26 1873553 10.1007/BF02633522

[bib55] Toniolo P, Levitz M, Zeleniuch-Jacquotte A, Banerjee S, Koenig KL, Shore RE, Strax P, Pasternack BS (1995) A prospective study of endogenous estrogens and breast cancer in postmenopausal women. J Natl Cancer Inst 87: 190–197 7707406 10.1093/jnci/87.3.190

[bib56] Valsta LM, Kilkkinen A, Mazur W, Nurmi T, Lampi AM, Ovaskainen ML, Korhonen T, Adlercreutz H, Pietinen P (2003) Phyto-oestrogen database of foods and average intake in Finland. Br J Nutr 89(Suppl 1): S32–S38 10.1079/BJN2002794 12725654

[bib57] Wang C, Mäkelä T, Hase T, Adlercreutz H, Kurzer M (1994) Lignans and flavonoids inhibit aromatase enzyme in human preadipocytes. J Steroid Biochem Mol Biol 50: 205–212 8049151 10.1016/0960-0760(94)90030-2

[bib58] Wu AH, Stanczyck FZ, Hendrich S, Murphy PA, Zhang C, Wan P, Pike MC (2000) Effects of soy foods on ovarian function in premenopausal women. Br J Cancer 82: 1879–1886 10839307 10.1054/bjoc.1999.1218 PMC2363237

[bib59] Zeleniuch-Jacquotte A, Adlercreutz H, Akhmedkhanov A, Toniolo P (1998) Reliability of serum measurements of lignans and isoflavonoid phytoestrogens over a two-year period. Cancer Epidemiol Biomarkers Prev 7: 885–889 9796633

[bib60] Zeleniuch-Jacquotte A, Shore RE, Koenig KL, Akhmedkhanov A, Afanasyeva Y, Kato I, Kim MY, Rinaldi S, Kaaks R, Toniolo P (2004) Postmenopausal levels of estrogen, androgen, and SHBG and breast cancer risk: long-term results of a prospective study. Br J Cancer 90: 153–159 14710223 10.1038/sj.bjc.6601517 PMC2395327

